# Foot orthoses: how much customisation is necessary?

**DOI:** 10.1186/1757-1146-2-23

**Published:** 2009-07-09

**Authors:** Hylton B Menz

**Affiliations:** 1 Musculoskeletal Research Centre, Faculty of Health Sciences, La Trobe University, Bundoora, Victoria, Australia

## Abstract

The relative merit of customised versus prefabricated foot orthoses continues to be the subject of passionate debate among foot health professionals. Although there is currently insufficient evidence to reach definitive conclusions, a growing body of research literature suggests that prefabricated foot orthoses may produce equivalent clinical outcomes to customised foot orthoses for some conditions. Consensus guidelines for the prescription of customised foot orthoses need to be developed so that the hypothesised benefits of these devices can be thoroughly evaluated.

## 

Those outside the foot health professions would be justifiably perplexed by the level of passion aroused by debates regarding the prescription and manufacture of foot orthoses. When all the professional turf wars, weekend workshops and marketing spin are stripped away, all that is left is a visually unremarkable piece of contoured thermoplastic placed inside the shoe. Despite technological advances which have significantly altered approaches to both the assessment of foot function and manufacture of foot orthoses [1,2], the devices themselves have actually changed surprisingly little over time. Nevertheless, it would take a very brave individual to claim that foot orthoses are nothing more than rebranded arch supports, particularly when an entire industry is sustained by the premise that modern foot orthoses are somehow different.

Part of the explanation for the passion associated with foot orthoses is that they do *appear *to be effective for a wide range of conditions [3], and there can be few experiences more satisfying to the foot health professional than alleviating a patient's chronic pain with foot orthoses when all other treatments have failed. In this context, clinicians can perhaps be forgiven for believing that their individual approach to the prescription and manufacture of the orthoses was responsible for the positive outcome, when in fact it is possible that a range of other approaches could have worked equally well.

Broadly speaking, it could be argued that there are two distinct approaches to the provision of foot orthoses – customised *versus *prefabricated – although these approaches are by no means mutually exclusive, as clinicians may interchangeably adopt either approach depending on individual patient needs and preferences. Furthermore, the distinction between customised and prefabricated orthoses has become somewhat blurred in recent years, with several custom orthoses laboratories offering generically contoured, semi-rigid orthoses in different sizes with a limited selection of shell modifications.

Nevertheless, the customised approach to foot orthosis provision is based on two main premises: (i) that clinical assessment can identify structural or functional deficits that may be contributing to the development of the presenting condition, and (ii) that the implementation of various design features into the manufacture of a foot orthosis (i.e. the "prescription") can selectively modify aspects of foot function, thereby alleviating symptoms. Proponents of prefabricated orthoses would argue that, with the exception of patients with marked morphological or functional abnormalities, the provision of a generically-contoured, prefabricated orthosis will sufficiently alter foot function to achieve equivalent clinical outcomes in most situations.

Notwithstanding the surprisingly common clinical anecdote regarding patients getting better despite wearing their orthoses upside down or in the wrong shoes, there is little doubt that in order to be comfortable, let alone effective, orthoses generally need to be an appropriate size and contour to approximate the morphology of the plantar surface of the foot. Where the two schools of thought start to diverge, however, is in relation to cast and shell modifications – a cornucopia of skives, grooves, wedges and cut-outs that some consider to be an essential component of the prescription.

This apparent dichotomy is by no means restricted to foot orthoses, as similar situations exist in many fields of healthcare. For example, in the physiotherapeutic treatment of low back pain, clinicians can be broadly categorized as "splitters" (i.e. those who believe that there are a wide range of anatomical causes of back pain which require detailed clinical assessment and targeted treatment) or "lumpers" (i.e. those who adopt the nomenclature of "non-specific low back pain" and approach treatment in a more generic, standardised manner) [4]. As with foot orthoses, the relative merits of these two approaches is the subject of passionate debate [5,6].

From a research evidence perspective, it is still too early to definitively conclude which approach to the provision of foot orthoses provides optimal clinical outcomes, however it is fair to say that the customised approach has experienced some setbacks in recent years. Not only have the theoretical frameworks and clinical assessment procedures commonly used to prescribe customised orthoses been seriously questioned [7-9], but several randomised controlled trials have shown prefabricated orthoses to have similar efficacy to customised orthoses in the management of plantar fasciitis [10-12], and, more recently, rheumatoid foot pain [13,14]. Although proponents of customised orthoses will invariably argue that the orthoses used in these trials were not optimally prescribed, such an argument is difficult to sustain in the absence of clearly defined, evidence-based prescription guidelines. Furthermore, those seeking solace in the conclusions of the recent Cochrane review of customised foot orthoses need to acknowledge that although there is good evidence for the effectiveness of customised devices for several conditions, the prescription protocols used in these trials were by no means consistent, and few trials used prefabricated orthoses as the comparator [15].

The June 2009 issue of *Journal of Foot and Ankle Research *contained an interesting paper by Redmond and colleagues [16] which reported the results of a biomechanical study comparing in-shoe plantar pressure patterns in 15 flat-footed participants wearing semi-rigid, customised orthoses and semi-rigid, prefabricated orthoses. Although both devices led to significant changes in pressure parameters compared to the shoe-only condition (primarily a shift of load from the forefoot and rearfoot toward the midfoot), there were no significant differences between the two devices. While acknowledging that recommendations regarding cost-effectiveness should be based on data from quality health economic studies, the authors nevertheless raised the issue of cost differences, stating that the custom devices were 2.5 times more expensive than the prefabricated devices, yet achieved very similar (biomechanical) outcomes.

The Redmond et al [16] study is not without its limitations. First, the sample was relatively small, so the lack of differences between the devices may have been due to type II error (i.e.: failing to observe a difference when in truth there is one). Secondly, the study was designed to examine biomechanical differences between the devices, rather than patient-oriented, clinical outcomes. Thirdly, the prefabricated orthoses were manufactured from the same materials as the customised devices (4 mm polypropylene shell with 450 kg/m^2 ^ethyl vinyl acetate heel posts), so the key differences being examined were the contour and frontal plane "correction" of the two orthoses. As such, the findings of this study cannot be generalised to other types of prefabricated orthoses that are commonly manufactured from more compliant materials. Finally, although the custom orthoses were "customised" in the sense that they were manufactured from a neutral impression cast and were posted to the individual's neutral calcaneal stance position (i.e.: the commonly employed "modified Root" technique), no additional cast or shell modifications were used.

Despite these limitations, the discomfiting question which arises from the Redmond et al [16] study it is this: Is there any substantial benefit to be gained from the additional time and resources required to perform an array of clinical measurements, take a plaster cast, write an individual prescription and have the devices individually manufactured, when selecting an appropriate prefabricated orthosis and simply placing it in the shoe may achieve very similar outcomes at far less cost? Neither the Redmond study nor those that have preceded it fully answer this question, but they do suggest that it is a question worth asking. Indeed, the burden of proof now sits squarely on the shoulders of proponents of customised orthoses, who need to justify why this additional activity and cost is necessary. This is a considerable challenge, as the essential first step to address this issue – the development of consensus guidelines for prescribing customised orthoses – will be difficult to achieve.

One way forward could be the application of the Delphi technique [17]. Based on the assumption that group judgements are more valid than individual judgements, this method is used to assist in reaching consensus agreement in areas where considerable variation of opinion exists. Briefly, this approach involves a facilitator asking a panel of experts a series of questions, the answers to which are then fed back to the group, and any common or conflicting viewpoints are identified. This process is repeated until opinions converge and a consensus is eventually reached. The Delphi technique has been successfully applied to solve problems in several fields of healthcare, including seemingly intractable topics such as the definition and classification of low back pain [18]. The development of consensus guidelines for the prescription of custom foot orthoses using such a technique would be a major step forward, and would provide a foundation upon which customised foot orthoses could be evaluated to the satisfaction of both researchers and clinicians.

Over time, further research may indeed reveal that there are subgroups of patients and conditions that respond more favourably to particular types of customised orthoses compared to prefabricated orthoses. However, given that many clinicians report high levels of success with orthotic therapy despite adopting a wide range of techniques, it is also possible that the individual prescription may not substantially contribute to the eventual outcome in many situations. While this proposition may be an affront to the more ardent proponents of customised foot orthoses, the average clinician may breathe a sigh of relief at the prospect of not having to perform an array of clinical measurements, take plaster casts, or fill in long orthotic prescription forms ever again. Nevertheless, we probably have a long way to go before the question posed by the title of this commentary can be satisfactorily answered.

## Competing interests

The author declares that they have no competing interests.

## References

[B1] Grumbine N (1993). Computer generated orthoses. A review. Clin Podiatr Med Surg.

[B2] Orlin M, McPoil T (2000). Plantar pressure assessment. Phys Ther.

[B3] Landorf K, Keenan A-M (2000). Efficacy of foot orthoses – what does the literature tell us?. J Am Podiatr Med Assoc.

[B4] Turk DC (2005). The potential of treatment matching for subgroups of patients with chronic pain: Lumping versus splitting. Clin J Pain.

[B5] McCarthy CJ, Cairns MC (2005). Why is the recent research regarding non-specific pain so non-specific?. Man Ther.

[B6] Wand BM, O'Connell NE (2008). Chronic non-specific low back pain – sub-groups or a single mechanism?. BMC Musculoskelet Disord.

[B7] McPoil TG, Hunt GC (1995). Evaluation and management of foot and ankle disorders: present problems and future directions. J Orthop Sports Phys Ther.

[B8] Payne CB (1998). The past, present and future of podiatric biomechanics. J Am Podiatr Med Assoc.

[B9] Nester CJ (2009). Lessons from dynamic cadaver and invasive bone pin studies: do we know how the foot really moves during gait?. J Foot Ankle Res.

[B10] Pfeffer G, Bacchetti P, Deland J, Lewis A, Anderson R, Davis W, Alvarez R, Brodsky J, Cooper P, Frey C, Herrick R, Myerson M, Sammarco J, Janecki C, Ross S, Bowman M, Smith R (1999). Comparison of custom and prefabricated orthoses in the initial treatment of proximal plantar fasciitis. Foot Ankle Int.

[B11] Martin JE, Hosch JC, Goforth WP, Murff RT, Lynch DM, Odom RD (2001). Mechanical treatment of plantar fasciitis. A prospective study. J Am Podiatr Med Assoc.

[B12] Landorf KB, Keenan AM, Herbert RD (2006). Effectiveness of foot orthoses to treat plantar fasciitis: a randomized trial. Arch Intern Med.

[B13] Novak P, Burger H, Tomsic M, Marincek C, Vidmar G (2009). Influence of foot orthoses on plantar pressures, foot pain and walking ability of rheumatoid arthritis patients-a randomised controlled study. Disabil Rehabil.

[B14] Cho NS, Hwang JH, Chang HJ, Koh EM, Park HS (2009). Randomized controlled trial for clinical effects of varying types of insoles combined with specialized shoes in patients with rheumatoid arthritis of the foot. Clin Rehabil.

[B15] Hawke F, Burns J, Radford JA, du Toit V (2008). Custom-made foot orthoses for the treatment of foot pain. Cochrane Database Syst Rev.

[B16] Redmond AC, Keenan AM, Landorf KB (2009). Contoured, prefabricated foot orthoses demonstrate comparable mechanical properties to contoured, customised foot orthoses: a plantar pressure study. J Foot Ankle Res.

[B17] Jones J, Hunter D (1995). Consensus methods for medical and health services research. BMJ.

[B18] Dionne CE, Dunn KM, Croft PR, Nachemson AL, Buchbinder R, Walker BF, Wyatt M, Cassidy JD, Rossignol M, Leboeuf-Yde C, Hartvigsen J, Leino-Arjas P, Latza U, Reis S, Gil Del Real MT, Kovacs FM, Oberg B, Cedraschi C, Bouter LM, Koes BW, Picavet HS, van Tulder MW, Burton K, Foster NE, Macfarlane GJ, Thomas E, Underwood M, Waddell G, Shekelle P, Volinn E, Von Korff M (2008). A consensus approach toward the standardization of back pain definitions for use in prevalence studies. Spine.

